# Horses Auto-Recruit Their Lungs by Inspiratory Breath Holding Following Recovery from General Anaesthesia

**DOI:** 10.1371/journal.pone.0158080

**Published:** 2016-06-22

**Authors:** Martina Mosing, Andreas D. Waldmann, Paul MacFarlane, Samuel Iff, Ulrike Auer, Stephan H. Bohm, Regula Bettschart-Wolfensberger, David Bardell

**Affiliations:** 1 Vetsuisse Faculty, University of Zurich, Zurich, Switzerland; 2 Swisstom AG, Landquart, Switzerland; 3 CTU Bern, University of Bern, Bern, Switzerland; 4 School of Veterinary Science, University of Liverpool, Liverpool, United Kingdom; 5 Veterinary University Vienna, Vienna, Austria; 6 Langford Veterinary Services, University of Bristol, Bristol, United Kingdom; University of Bari, ITALY

## Abstract

This study evaluated the breathing pattern and distribution of ventilation in horses prior to and following recovery from general anaesthesia using electrical impedance tomography (EIT). Six horses were anaesthetised for 6 hours in dorsal recumbency. Arterial blood gas and EIT measurements were performed 24 hours before (baseline) and 1, 2, 3, 4, 5 and 6 hours after horses stood following anaesthesia. At each time point 4 representative spontaneous breaths were analysed. The percentage of the total breath length during which impedance remained greater than 50% of the maximum inspiratory impedance change (breath holding), the fraction of total tidal ventilation within each of four stacked regions of interest (ROI) (distribution of ventilation) and the filling time and inflation period of seven ROI evenly distributed over the dorso-ventral height of the lungs were calculated. Mixed effects multi-linear regression and linear regression were used and significance was set at p<0.05. All horses demonstrated inspiratory breath holding until 5 hours after standing. No change from baseline was seen for the distribution of ventilation during inspiration. Filling time and inflation period were more rapid and shorter in ventral and slower and longer in most dorsal ROI compared to baseline, respectively. In a mixed effects multi-linear regression, breath holding was significantly correlated with PaCO_2_ in both the univariate and multivariate regression. Following recovery from anaesthesia, horses showed inspiratory breath holding during which gas redistributed from ventral into dorsal regions of the lungs. This suggests auto-recruitment of lung tissue which would have been dependent and likely atelectic during anaesthesia.

## Introduction

In standing horses lung perfusion and ventilation are concentrated in the caudo-dorsal regions of the lungs [1, 2], leading to a nearly perfect ventilation/perfusion (V/Q) match, with less than 3% shunt fraction [3].

During anaesthesia in lateral and dorsal recumbency, ventilation-perfusion relationships alter with perfusion preferentially concentrated in dorsal and dependent lung regions, whilst ventilation is preferentially directed to non-dependent lung regions [4]. Intrapulmonary shunt fraction increases to > 30% of cardiac output in ponies in dorsal recumbency due to atelectasis formation within 30 minutes of anaesthesia [3, 5]. Little is known regarding resolution of these ventilation- perfusion disturbances following recovery from anaesthesia, but in the authors’ experience alterations in respiratory rate, effort and cycle duration can frequently be observed in horses after recovery from anaesthesia, possibly reflecting a compensatory mechanism to counteract persistent atelectasis.

Electrical impedance tomography (EIT) is a novel method of investigating and monitoring regional lung function. For this purpose, 32 electrodes are placed around the thorax, weak alternating currents are applied sequentially via two of these electrodes and the resulting potentials are measured at the remaining electrodes. From the measured voltages, regional impedance changes are calculated and sequences of real-time images generated, representing organ function rather than structure [6]. It has been shown that intra-thoracic impedance changes with ventilation [7–10] and the cardiac cycle [11], with EIT signals due to ventilation being approximately 10 times the magnitude of those related to cardiac function and pulmonary perfusion.

The aim of this study was to evaluate the breathing pattern, distribution of ventilation and gas exchange in horses for 6 hours following recovery from a 6 hour period of anaesthesia, with reference to their pre-anaesthetic values, using EIT. Our hypothesis was that persistent atelectasis in dorsal lung regions caused a redirection of ventilation to more ventral lung regions following recovery from anaesthesia.

## Materials and Methods

Following ethical approval by the Federal Food Safety and Veterinary Office FSVO of the Swiss government (Reference TV-4985), eight healthy adult horses with a mean (±SD) age of 10.0 ± 5.5 years and body mass 538 ± 36 kg were included in this study. All horses were judged healthy based on clinical examination with particular emphasis on respiratory evaluation, routine haematology and biochemistry. Two horses underwent anaesthesia one week before the experiment as part of a separate study [12].

### Animal preparation before anaesthesia

Baseline (BL) arterial blood samples were taken 24 hours before anaesthesia by direct puncture of the carotid artery and analysed immediately for oxygen and carbon dioxide tensions (PaO_2_ and PaCO_2_ respectively) (Rapidpoint, Siemens, Germany).

A narrow circumferential strip of hair was clipped around the thorax directly caudal to the scapula (5^th^- 6^th^ intercostal space) to aid consistent positioning of the electrical impedance belt. Electrically non-conductive ultrasound gel was applied to the clipped area and an EIT belt was placed under slight tension around the thorax. The belt was made of an elastic rubber tube on which 32 stainless steel contacts of 1 cm^2^ were mounted equidistantly with the 1^st^ and 32^nd^ electrode being placed ventrally over the sternum. A surcingle was placed over the EIT belt to ensure all electrodes were in firm contact with the skin.

EIT measurements were performed for a period of 2 minutes or longer if at least 4 consecutive breaths without any artefacts (due to gross movement or muscle fasciculations) had not been recorded within this time, with the horse standing quietly and breathing regularly.

### Anaesthesia

A pulmonary artery catheter was placed via the jugular vein under local anaesthesia, prior to administration of pre-anaesthetic medication. Standardised anaesthetic and monitoring protocols were followed and were defined by the requirements of a separate study [12]. Pre-anaesthetic medication consisted of medetomidine (Dorbene, Graub AG, Switzerland) 0.007 mg/kg IV and phenylbutazone (Butadion, Streuli Pharma AG, Switzerland) 4.4 mg/kg IV. Induction of anaesthesia with diazepam (Valium, Roche, Switzerland) 0.02 mg/kg IV and ketamine (Ketanarkon 100, Streuli Pharma AG, Switzerland) 2 mg/kg IV preceded endotracheal intubation. Horses were subsequently positioned in dorsal recumbency, the endotracheal tube connected to a large animal circle breathing system (Tafonius, Vetronic Services Ltd, UK) and anaesthesia maintained for 6 hours using isoflurane (Attane Isoflurane, Provet A, Switzerland) vaporised in an oxygen/air mix (fraction inspired oxygen 0.5) and medetomidine constant rate intravenous infusion (0.0035 mg/kg/hr). The facial artery was cannulated to enable invasive monitoring of arterial blood pressure, samples to be taken for blood gas analysis and cardiac output (Qt) measurement using the lithium dilution technique (LiDCO, LiDCO Group, UK) [13] during the anaesthetic period. All arterial blood samples were collected anaerobically into 2.5 mL pre-heparinised syringes (BD Preset, Becton Dickinson, USA), after first discarding 5 mL of blood, and analysed immediately.

Morphine (0.1 mg/kg IV) was infused using a syringe pump over the last 30 minutes of anaesthesia and arterial and mixed venous blood gas samples were analysed at the end of anaesthesia to enable calculation of intrapulmonary shunt. Thereafter isoflurane delivery was discontinued and horses allowed to recover unassisted in a padded recovery box.

### Data collection after recovery

One, 2, 3, 4, 5 and 6 hours (t1-t6) after standing, EIT measurements were repeated and arterial blood samples collected and analysed as described above.

### Data and EIT data analysis

Venous admixture (Qs/Qt) at the end of anaesthesia was calculated retrospectively using Berggren’s equation [14]. EIT measurements were performed with a modified Pioneer-Set (Swisstom AG, Switzerland). The system produces 46 real time images per second and is described elsewhere [15]. Images were calculated using a Graz consensus reconstruction algorithm for EIT (GREIT) [16, 17] and represent a cross-section of the thorax with a slice thickness of approximately 10% of the diameter [18]. Regional impedance changes (ΔZ_r_) were converted to pixel values and displayed in functional images as grey values. Changes in thoracic impedance have been shown to be proportional to local changes of air content within the respective lung region [19]. A finite element equine mesh was used for EIT image reconstruction. The global impedance time curve ΔZ_g_(*t*) was calculated as the sum of the impedance change of all lung pixels. Respiratory rate (RR) and tidal volume (VT_EIT_) were calculated using the impedance change of the EIT signal.

**Fig 1:**
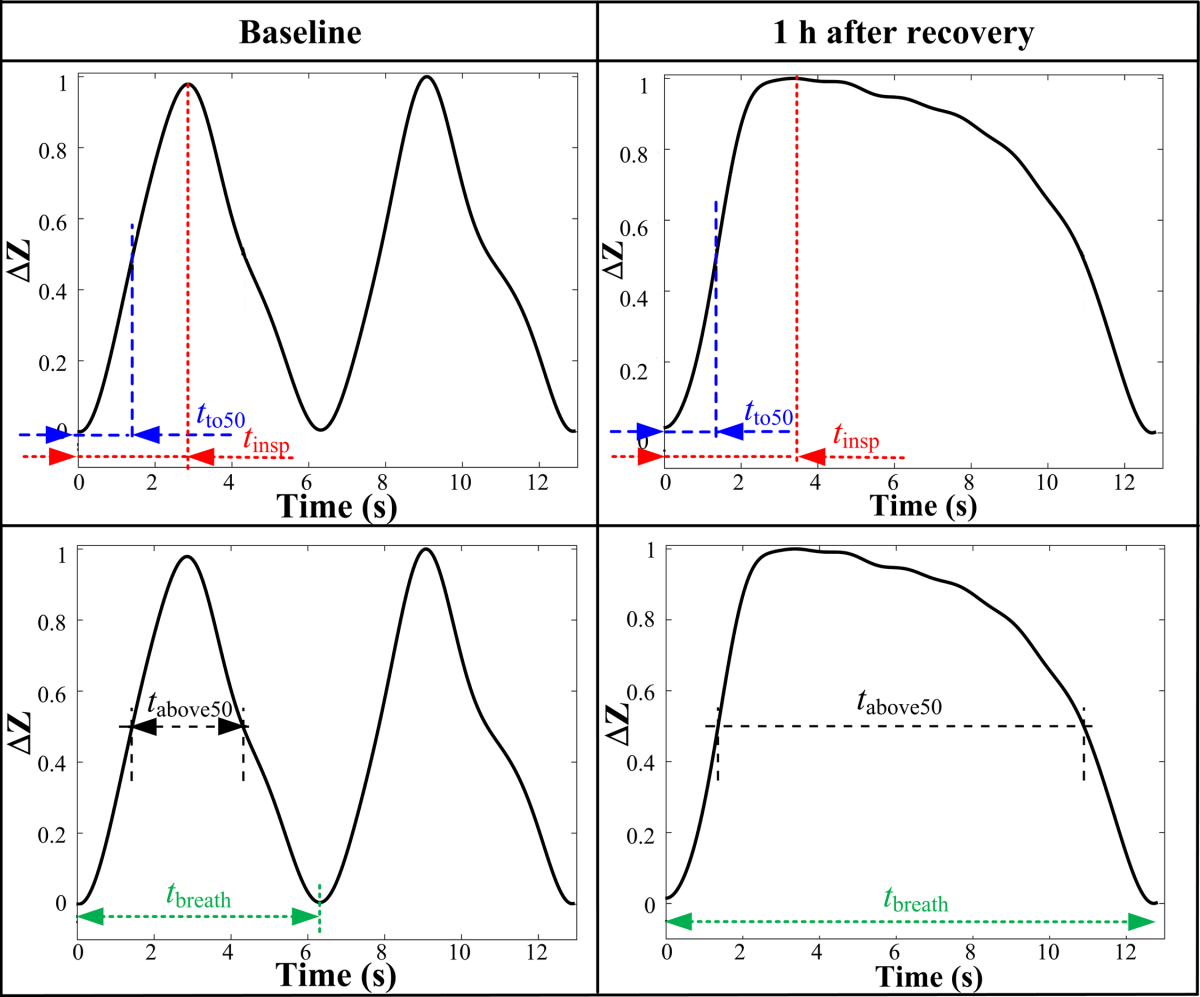
Curve of change in impedance (ΔZ) over the respiratory cycle of a representative horse 24 hours before anaesthesia (baseline) and one hour after recovering from anaesthesia. Breath holding is defined as the percentage of the total breath length (*t*_breath_) in which impedance remained higher than 50% (*t*_above50_) of the maximum change in impedance during inspiration. Inspiratory time = *t*insp; time to reach 50% of maximum impedance change during inspiration = *t*to50.

At each time point, 4 representative breaths were analysed from the EIT recording. The percentage of the total breath length (*t*_breath_) during which impedance remained higher than 50% of maximum inspiratory impedance change (*t*_above50_) was calculated, and defined to represent the time of breath holding (Fig 1).

**Fig 2:**
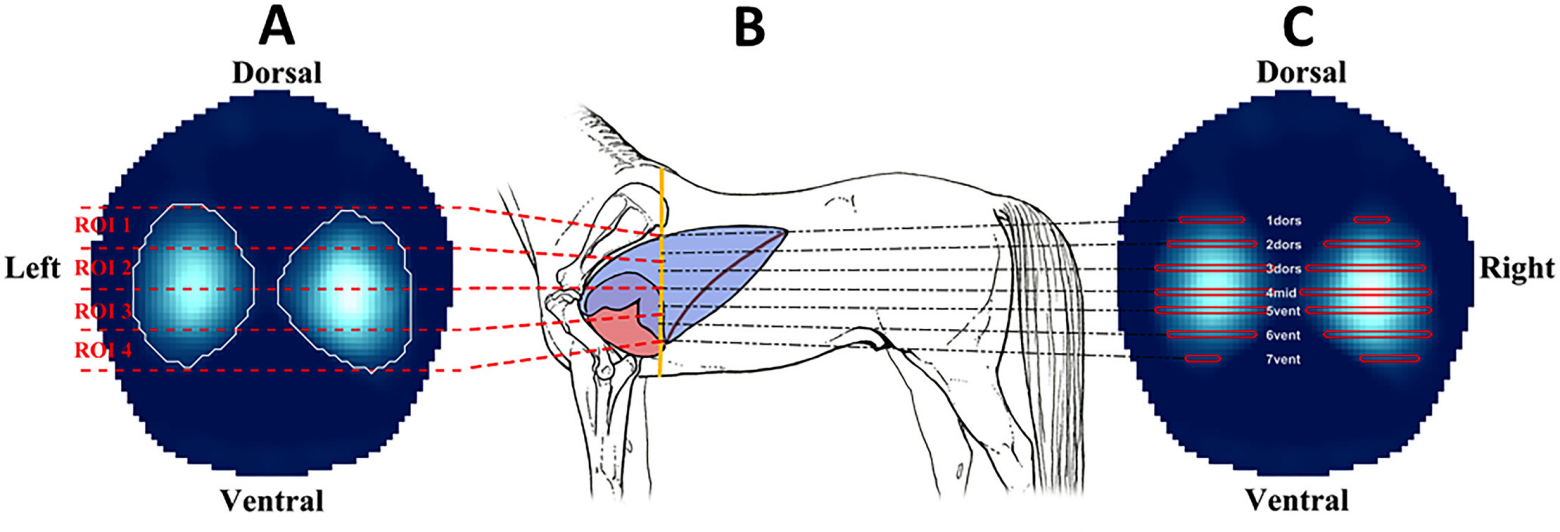
**Illustration showing the four stacked regions of interest (ROI) for evaluating dorso-ventral distribution of ventilation during inspiration (A), the belt position on the thorax (B) and the 7 ROI symmetrically distributed over the height of the lung field to determine regional filling times and inflation periods (C)**.

To describe the dorso-ventral distribution of ventilation during inspiration, the approach described by Radke et al. (2012) was adapted [20]. To analyse the total tidal ventilation distribution at baseline, the EIT generated image was divided into four stacked regions of interest (ROI1 –ROI4), with ROI1 being the most dorsal and ROI4 the most ventral regions (Fig 2A). Distribution of ventilation within these pre-defined ROI after recovery from anaesthesia was then compared to that at baseline.

**Fig 3:**
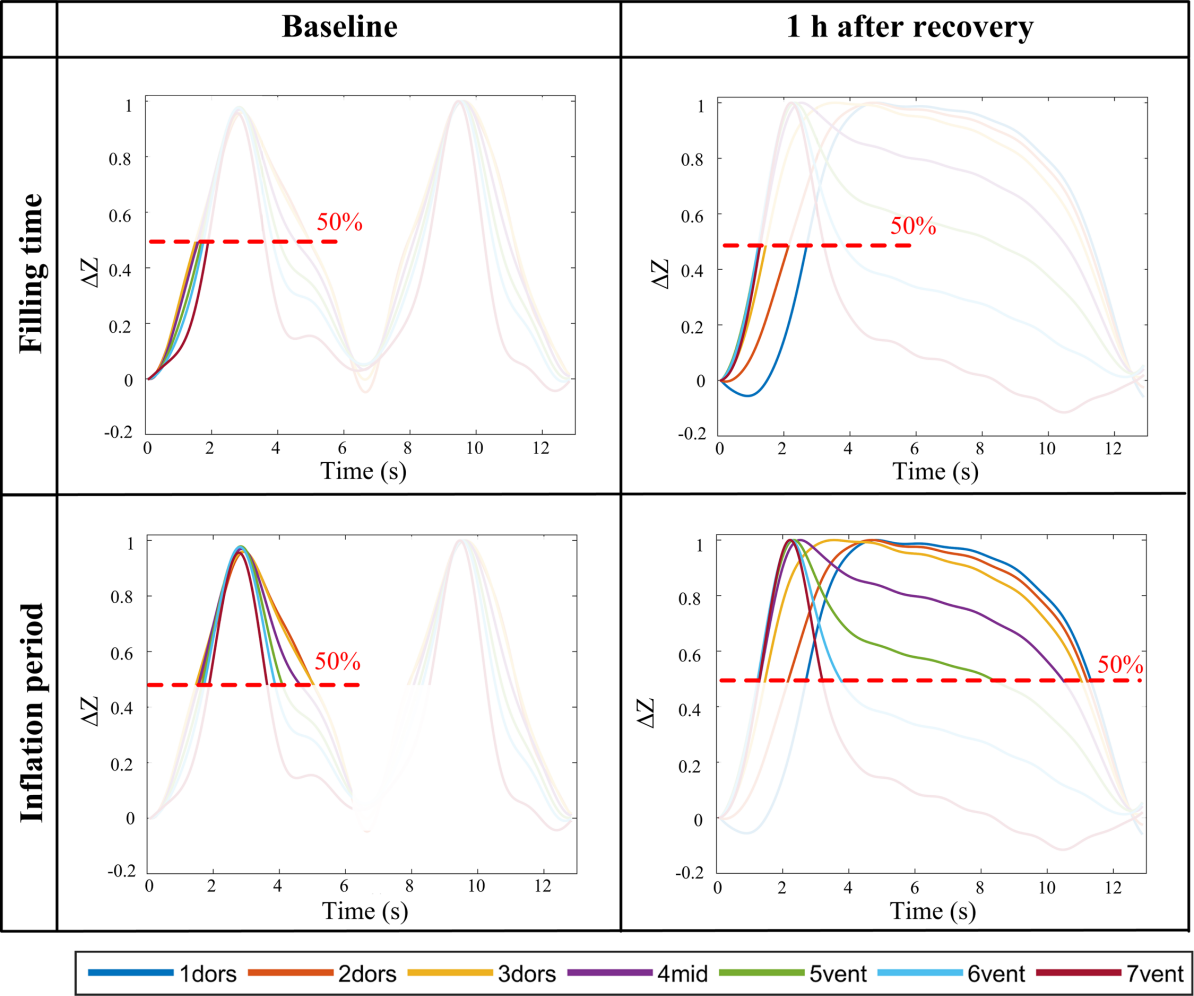
Regional time delays of 7 regions of interest (ROI) to evaluate filling time and inflation period. Filling times were normalised by inspiration time; inflation periods were normalised by total breath length. Curves for both variables were normalized by the amplitude. ROIs were defined as 3 dorsal (1_dors_, 2_dors_ 3_dors_), one middle (4_mid_) and 3 ventral (5_vent_, 6_vent_, 7_vent_) ROI distributed evenly over the total height of the lung field. Figure illustrates examples from representative horse at baseline and 1 hour after recovery from anaesthesia.

To analyse regional time delays within the lung seven ROI (1_dors_, 2_dors_, 3_dors_, 4_mid_, 5_vent_, 6_vent_, 7_vent_) were defined over the dorsal-ventral height of the lung field (Fig 2C). Each ROI represented a horizontal section one pixel wide, with ROI 4_mid_ defining the mid-point dorso-ventrally. The lung fields dorsal and ventral to this were further subdivided equally into upper, mid and lower regions. Two time characteristics were evaluated:

1. Regional filling time (*t*fill_r_) = *t*to50_r_ / *t*insp_g_. The time at which ΔZ_r_(*t*) of each region reached 50% of its maximum impedance change during inspiration (*t*to50_r_), normalised by the global inspiratory time (*t*insp_g_). The start of inspiration was defined in the global impedance curve (Fig 3).
2. Regional inflation period (*t*infl_r_) = *t*above50_r_ / *t*breath_g_. The time period during which ΔZ_r_(*t*) of each lung region remained above 50% (*t*above50_r_) of its maximum inspiratory impedance change, normalised by the global breath length (*t*breath_g_) (Fig 3).

### Statistical analysis

For continuous variables, ANOVA was used to compare all variables during the different measurement time points. Levene's test of heterogeneity of variance was used beforehand. A mixed effects multi-linear model was used fitting the model via maximum likelihood. To show the influence of physiological variables on our primary endpoint of breath holding, a multivariate mixed effects regression was used and the independent variables RR, PaCO_2_, PaO_2_ and VT_EIT_ were tested. First, univariate models were developed, followed by multivariable-adjusted analyses. Using a step-wise backward elimination process the least significant variables were removed from the base model. Only variables with p<0.05 remained in the final model. The random effect variables were kept in all models.

A second analysis was run to estimate the time for return to baseline. A linear regression model was applied for time points 1 to 6 after anaesthesia and visually checked for significant difference, using the 95% CI of the baseline measurements and the corresponding 95% CI of the linear regression of the measurements after baseline. Return to baseline was assumed when either the lower baseline CI crossed the upper CI of the linear regression or vice versa.

## Results

Data from two horses were excluded. One horse sustained a carpal wound during recovery from anaesthesia, requiring sedation for wound management and technical difficulties with the EIT system were encountered with one horse. Arterial blood samples and EIT measurements were obtained from the remaining 6 horses at the pre-determined time-points and data from these six horses were evaluated.

At the end of six hours of anaesthesia, two horses were mechanically ventilated (controlled mechanical ventilation initiated after 3 and 3.5 hours of anaesthesia) and four breathed spontaneously (three horses on continuous positive airway pressure (CPAP) 8 cmH_2_O; one horse on atmospheric airway pressure). Mean ± SD of the venous admixture of the three mechanically ventilated and spontaneously breathing horses at the end of anaesthesia was 25.7 ± 21.4 and 26.3 ± 8.2%, respectively. The time from end of anaesthesia until standing was 76 ± 30 minutes (mean ± SD).

Results of the EIT findings and blood gas analyses are summarised in Table 1.

**Table 1:** Results of EIT and arterial blood gas measurements (mean ± SD) in 6 horses for the first 6 hours (t1-t6) after recovery from anaesthesia. ROI = Region of interest 1–4; *t*fill_r_ = Filling time for each region (1_dors_, 2_dors_, 3_dors_, 4_mid_, 5_vent_, 6_vent_, 7_vent_); *t*infl_r_ = Inflation period for each region (1_dors_-7_vent_); RR = Respiratory rate; VT_EIT_ = Tidal volume as calculated from the EIT signal; PaCO_2_ = Arterial partial pressure of carbon dioxide; PaO_2_ = Arterial partial pressure of oxygen. Values are percentages unless otherwise stated.

	Baseline	t1	t2	t3	t4	t5	t6	p Value
hold %	45.7 ± 9.0	67.5 ± 8.6	62.1 ± 10.8	55.5 ± 6.2	56.0 ± 8.0	53.1 ± 8.3	52.0 ± 8.4	<0.001
ROI 1	13.0 ± 3.8	10.2 ± 6.2	13.2 ± 3.0	12.0 ± 4.9	12.5 ± 5.3	11.5 ± 4.3	10.4 ± 3.8	0.202
ROI 2	36.6 ± 3.5	35.3 ± 5.7	36.2 ± 4.5	36.0 ± 5.9	36.4 ± 3.9	36.6 ± 2.4	34.1 ± 1.7	0.353
ROI 3	38.9 ± 3.4	41.9 ± 4.8	40.1 ± 2.5	41.1 ± 6.0	40.0 ± 4.6	40.6 ± 3.4	41.7 ± 3.2	0.176
ROI 4	11.5 ± 2.6	12.6 ± 6.8	10.5 ± 4.9	10.8 ± 6.5	11.2 ± 4.7	11.3 ± 2.4	13.7 ± 2.8	0.250
*t*fill_r_ 1_dors_	49.5 ± 9.2	48.5 ± 22.7	56.1 ± 15.1	49.5 ± 13.3	45.2 ± 16.6	47.6 ± 15.3	48.5 ± 8.3	0.511
*t*fill_r_ 2_dors_	51.3 ± 4.9	52.3 ± 10.1	53.5 ± 8.6	51.6 ± 10.3	48.6 ± 11.5	50.6 ± 9.4	51.6 ± 7.7	0.735
*t*fill_r_ 3_dors_	52.2 ± 3.8	47.4 ± 5.5	50.3 ± 3.4	48.1 ± 13.4	48.5 ± 8.5	49.8 ± 6.6	52.3 ± 8.3	0.190
*t*fill_r_ 4_mid_	52.8 ± 3.9	43.9 ± 7.1	48.2 ± 4.7	47.6 ± 12.1	48.6 ± 7.5	49.5 ± 5.9	50.4 ± 6.3	0.003
*t*fill_r_ 5_vent_	53.5 ± 4.1	41.7 ± 8.0	47.0 ± 6.4	46.3 ± 12.0	48.7 ± 8.9	49.5 ± 5.6	49.6 ± 6.1	<0.001
*t*fill_r_ 6_vent_	53.9 ± 4.8	40.8 ± 11.1	45.0 ± 7.0	46.7 ± 6.6	47.6 ± 9.7	49.0 ± 6.5	49.1 ± 6.0	<0.001
*t*fill_r_ 7_vent_	53.3 ± 7.6	37.9 ± 12.9	37.1 ± 22.6	36.4 ± 23.5	39.7 ± 20.8	47.3 ± 9.0	47.7 ± 11.5	0.002
*t*infl_r_ 1_dors_	54.7 ± 10.4	57.8 ± 18.4	63.2 ± 13.3	52.8 ± 13.1	58.2 ± 13.3	49.5 ± 15.7	54.7 ± 12.1	0.086
*t*infl_r_ 2_dors_	51.6 ± 7.2	66.5 ± 6.5	62.5 ± 10.6	54.4 ± 9.8	58.7 ± 10.8	52.8 ± 8.5	56.6 ± 7.4	<0.001
*t*infl_r_ 3_dors_	48.7 ± 7.8	66.3 ± 8.3	61.0 ± 9.7	53.8 ± 11.5	58.3 ± 10.9	52.8 ± 7.9	54.8 ± 7.0	<0.001
*t*infl_r_ 4_mid_	46.6 ± 7.9	65.3 ± 9.5	59.3 ± 9.5	53.0 ± 13.0	55.7 ± 9.7	52.7 ± 8.7	53.0 ± 7.4	<0.001
*t*infl_r_ 5_vent_	44.1 ± 7.6	57.9 ± 11.2	54.1 ± 10.0	51.3 ± 10.6	51.6 ± 10.7	50.5 ± 9.6	48.9 ± 9.4	<0.001
*t*infl_r_ 6_vent_	41.2 ± 8.3	45.1 ± 16.8	44.2 ± 18.9	47.6 ± 11.2	48.3 ± 12.5	48.7 ± 11.4	43.8 ± 9.6	0.366
*t*infl_r_ 7_vent_	37.1 ± 9.4	34.3 ± 15.0	37.7 ± 28.6	36.9 ± 19.0	35.1 ± 17.3	43.6 ± 12.4	39.7 ± 8.7	0.503
RR (breaths per minute)	7.6 ± 0.8	6.0 ± 2.9	7.1 ± 2.0	9.2 ± 2.2	8.8 ± 3.6	8.9 ± 2.3	10.1 ± 4.3	0.247
VT_EIT_ (mL)	6079.7 ± 2733.3	5120.3 ± 1631.7	5355.8 ± 1987.6	5258.2 ± 1856.6	5058.3 ± 2413.0	5086.1 ± 1617.1	5308.5 ± 1989.9	0.982
PaCO_2_ kPa (mmHg)	5.6 ± 0.4 (42.0 ± 3.2)	6.2 ± 1.2 (46.7 ± 8.7)	6.6 ± 0.5 (49.5 ± 3.4)	5.9 ± 0.8 (43.9 ± 5.7)	5.7 ± 0.3 (42.4 ± 2.0)	5.2 ± 0.3 (39.0 ± 2.0)	5.3 ± 0.3 (39.4 ± 2.5)	0.004
PaO_2_ kPa (mmHg)	12.0 ± 1.2 (90.0 ± 9.2)	11.1 ± 1.6 (83.2 ± 12.0)	10.9 ± 1.5 (82.0 ± 11.0)	11.1 ± 1.9 (83.2 ± 14.1)	10.3 ± 1.5 (77.5 ± 11.5)	11.2 1.3 (84.2 ± 9.9)	11.0 ± 1.2 (82.2 ± 9.2)	0.840

**Fig 4:**
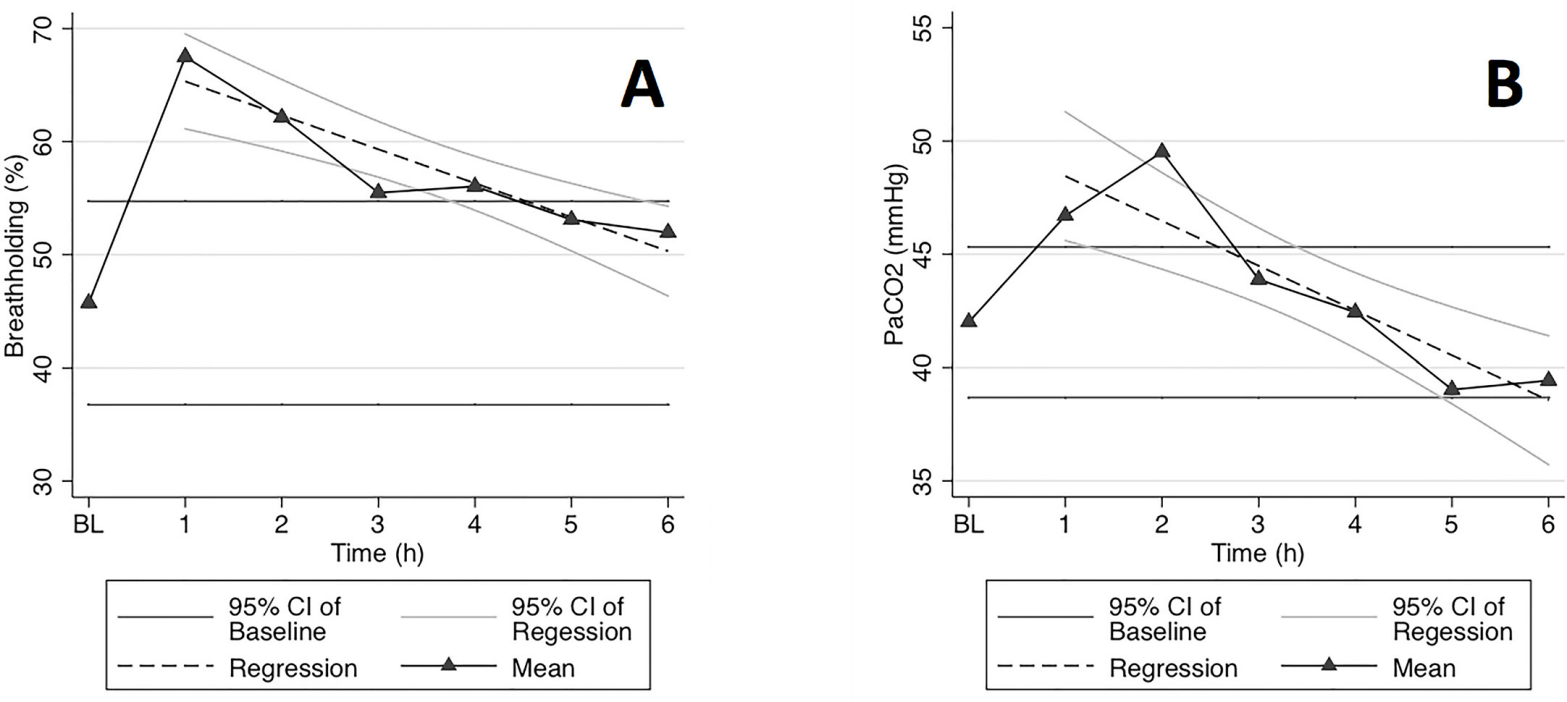
**Graphs of (A) breath holding as a percentage (%) of total breath length and (B) PaCO_2_ (mmHg) as measured at baseline (BL) and for 6 hours after recovery from general anaesthesia**.

Breath holding could be demonstrated in all horses and was seen until 5 hours after standing (Fig 4).

No significant change from baseline could be shown for any of the four stacked ROI for the dorso-ventral distribution of ventilation during inspiration.

While the filling time remained unchanged in the two most dorsal ROI (1_dors_ and 2_dors_), it was significantly shortened in all other ROI. Changes in ROI 3_dors_ were less prominent and seen until 3 hours after standing. A significant decrease in filling time was seen in the middle and 3 ventral ROI until 5 hours after standing.

The inflation period was unchanged in ROI 1_dors_, 6_vent_ and 7_vent_, but was prolonged in ROI 2_dors_, 3_dors_, 4_mid_ and 5_vent_ compared to baseline (Fig 3).

The mixed effects model showed that PaCO_2_ and time point remained significant for breath-holding in the final model (p<0.003). Effects were not found to be significant for VT_EIT_, RR and PaO_2_ on breath-holding in the multivariate regression.

Respiratory rate increased over time and was significantly higher after t5. VTEIT did not change significantly compared to baseline. None of the horses showed a PaO_2_ < 8 kPa at any time point after recovery (Table 1).

## Discussion

To the authors’ knowledge this is the first description of the phenomenon of inspiratory breath holding in horses after recovery from general anaesthesia. Additionally we have identified that gas redistributes from ventral to dorsal regions of the lung during this period.

In the authors’ experience, breath holding can be observed regularly in the quietly standing horse after recovery from anaesthesia. Electrical impedance tomography provided a safe, non-invasive tool to investigate this phenomenon and to determine, by repeated measurements, for how long after anaesthesia ventilation dynamics remained altered.

Beside the evaluation of the global ΔZ_g_(*t*) signals, the main feature of EIT is the capability to measure the spatial distribution of ventilation within the thoracic plane defined by the circumferential electrodes. A change in dorso-ventral distribution of ventilation during inspiration into the ventral ROI was expected after anaesthesia as our horses were positioned in dorsal recumbency and venous admixture values of 20–30% were calculated at the end of anaesthesia. This is consistent with formation of significant atelectasis, likely in dependent lung regions as previously described during anaesthesia in horses [5]. This was not confirmed, however, and no differences in dorso-ventral distribution of ventilation during inspiration were found at any time point after recovery from anaesthesia. This observation is consistent with the situation in humans where no change in dorso-ventral distribution of ventilation during inspiration was observed after anaesthesia of up to 12 hours duration [20, 21].

Dynamic EIT images (S1 and S2 Files (Baseline impedance changes with respiratory cycle and Impedance changes with respiratory cycle after recovery from anaesthesia)) demonstrated regional impedance changes within each breathing cycle consistent with an unexpected redistribution of gas in the opposite direction from ventral into dorsal lung regions during breath holding. To verify this subjective observation, two previously described variables were adapted representing the filling and inflation period of specifically defined ROI [22]. Seven ROI were defined, equally distributed dorso-ventrally over the lung field. Subdividing dorsal and ventral lung fields in this way confirmed our subjective observation of gas redistribution within the lung and retained sufficient sensitivity to show a good spatial distribution and the linearity of the redistribution. The independent analysis of the seven ROI showed more rapid filling and shorter inflation of ventral regions, whilst filling and inflation of the dorsal ROI was delayed and prolonged except 1_dors_ following recovery from anaesthesia. This is consistent with emptying of ventral into dorsal regions during the breath holding period, suggesting auto-recruitment of atelectic lung tissue in dorsal regions which had been dependent during the anaesthetic period. The phenomenon of auto-recruitment by breath holding has not been described previously and may be unique to horses.

Several distinct breathing patterns have been described in horses including ‘big respiratory cycles (BRC) [23, 24] during and after maximum exercise and ‘deep sighing’ in the postoperative period [25]. In anaesthetised ponies a long inspiratory pause due to laryngeal closure, confirmed by laryngoscopy, has been described, which occurred as soon as consciousness was lost, regardless of the anaesthetic agent used [26]. Laryngeal braking is described in human infants and causes an elevation in end-expiratory reserve volume [27]. Laryngeal braking showing the same flow pattern as that described in ponies has also been recorded in preterm human infants [28], being triggered by steady-state inhalation of 2% CO_2_ in the absence of a reduction in SpO_2_. The effect of provoked hypercapnia on laryngeal muscles was verified by electromyographic readings. A parallel increase of PaCO_2_ and breath holding without hypoxaemia was observed in our horses. While it remains unclear whether the high PaCO_2_ was caused by the breath holding, or the reason for it, the comparable trend over time of these two variables and the findings in infants with the similarity in flow pattern is an indication that the observed breath holding may be triggered by hypercapnia due to laryngeal braking or closure.

EIT has been used in ponies during and after pregnancy [29]. The feasibility of repeated measurements was demonstrated by evaluating distribution of ventilation by splitting the EIT image into four slices from dorsal to ventral. As in our horses at baseline, ventilation was equally distributed between the two dorsal and two ventral ROI. To allow the analysis of local redistribution of ventilation during each breath we performed a breath-by-breath analysis while in the aforementioned paper the root mean square values of tidal impedance changes over five consecutive breath was calculated.

Spirometry could be used as an alternative method for evaluating the breathing pattern in horses before and after anaesthesia. This however would require a greater degree of restraint during measurements which may influence the behaviour and therefore breathing pattern of the subject. Alternatively, respiratory ultrasonic plethysmography could be used as signals are transmitted via Bluetooth [30]. Neither of these methods however provide any insights into the regional distribution of tidal gas movement within the thorax. All our horses tolerated the EIT device well and measurements could be performed with the horse standing quietly and unrestrained in a stable.

One possible explanation for the observed breath holding might have been the administration of morphine at the end of anaesthesia. This however is very unlikely as morphine has been administered at the same dose intravenously to conscious and anaesthetised horses without any reported effect on respiratory rate or PaCO_2_ [31, 32].

### Limitations and future studies

The major limitation of this study was the identification and recording of representative breaths for analysis. This was due to the behaviour of the horses over the period of investigation, such as movement artefacts from muscle fasciculations, interactions with the investigator, sniffing, scratching, rubbing, eating or moving around the box. We arbitrarily chose to define four consecutive artefact free breath recordings as our criteria for successful recording and analysis. Whilst this meant that in some instances we had to extend the recording period for longer than two minutes, this was always achievable within ten minutes. Restraining the horses during the recording periods would have been an alternative but we elected to minimise our interference with the horses’ normal behaviour, assuming this would result in more natural breathing patterns. The difference in modes of ventilation at the end of anaesthesia adds a confounding factor to the comparison of the lung status after recovery, particularly as we only studied six horses. We were able, however, to demonstrate the same breath holding phenomenon in all six horses and the different ventilation strategies reflect those employed in clinical anaesthetic management.

Furthermore, statistical analysis of the degree of venous admixture at end of anaesthesia did not appear to influence the duration of breath holding at t1. In humans the use of spontaneous breathing versus controlled mechanical ventilation during anaesthesia had no influence on the distribution of ventilation after anaesthesia [20]. Larger studies are warranted to investigate the influence of different lung states after anaesthesia on the breath holding phenomenon.

Two horses underwent general anaesthesia as part of a separate research project one week prior to this study. Whilst we cannot rule out a residual effect on lung function from the first anaesthetic period on our results, baseline arterial blood gas analysis obtained prior to the second anaesthetic was not different to that obtained prior to the first anaesthetic event. Additionally baseline arterial blood gas analysis, clinical examination and EIT recordings obtained from these individuals were not significantly different from those obtained from the other horses.

Future studies need to investigate the relationship between elevated PaCO_2_ levels and breath holding and whether laryngeal braking or closure is indeed the mechanism. This can only be achieved by using EIT in combination with spirometry or respiratory ultrasonic plethysmography and laryngoscopy.

In conclusion, this study describes for the first time, that after an extended anaesthetic period horses performed inspiratory breath holding. During this breath holding period gas redistributed from ventral to dorsal regions of the lungs, corresponding to an auto-recruitment of collapsed lung tissue within the perioperatively dependant dorsal lung regions. Similar trends over time were observed for both PaCO_2_ and breath holding, but future studies need to further evaluate the relationship between these two variables.

## Supporting Information

S1 File S1.File.avi. **Baseline impedance changes with respiratory cycle.** Regions of higher impedance, consistent with increasing gas content of lung tissue are denoted by paler colouration during the respiratory cycle.
(AVI)

S2 File S2.File.avi. **Impedance changes with respiratory cycle after recovery from anaesthesia.** Note the increase in duration of increased intrathoracic impedance consistent with the breath holding phenomenon and also the change in distribution of impedance within the thorax during the breath holding period.
(AVI)
